# Genetic modification of Gγ subunit AT1 enhances salt-alkali tolerance in main graminaceous crops

**DOI:** 10.1093/nsr/nwad075

**Published:** 2023-03-23

**Authors:** Wenjing Sun, Huili Zhang, Sen Yang, Lijing Liu, Peng Xie, Jian Li, Yaoyao Zhu, Yidan Ouyang, Qi Xie, Huawei Zhang, Feifei Yu

**Affiliations:** National Key Laboratory of Wheat Improvement, Peking University Institute of Advanced Agricultural Sciences, Shandong Laboratory of Advanced Agriculture Sciences in Weifang, China; State Key Laboratory of Plant Genomics, Institute of Genetics and Developmental Biology, The Innovative Academy of Seed Design, Chinese Academy of Sciences, China; State Key Laboratory of Plant Genomics, Institute of Genetics and Developmental Biology, The Innovative Academy of Seed Design, Chinese Academy of Sciences, China; The Key Laboratory of Plant Development and Environmental Adaptation Biology, Ministry of Education, School of Life Sciences, Shandong University, China; State Key Laboratory of Plant Genomics, Institute of Genetics and Developmental Biology, The Innovative Academy of Seed Design, Chinese Academy of Sciences, China; National Key Laboratory of Wheat Improvement, Peking University Institute of Advanced Agricultural Sciences, Shandong Laboratory of Advanced Agriculture Sciences in Weifang, China; National Key Laboratory of Wheat Improvement, Peking University Institute of Advanced Agricultural Sciences, Shandong Laboratory of Advanced Agriculture Sciences in Weifang, China; National Key Laboratory of Crop Genetic Improvement and National Centre of Plant Gene Research (Wuhan), Hubei Hongshan Laboratory, Huazhong Agricultural University, China; State Key Laboratory of Plant Genomics, Institute of Genetics and Developmental Biology, The Innovative Academy of Seed Design, Chinese Academy of Sciences, China; National Center of Technology Innovation for Maize, State Key Laboratory of Maize Germplasm Innovation and Molecular Breeding, Syngenta Group China, China; University of Chinese Academy of Sciences, China; National Key Laboratory of Wheat Improvement, Peking University Institute of Advanced Agricultural Sciences, Shandong Laboratory of Advanced Agriculture Sciences in Weifang, China; College of Grassland Science and Technology, China Agricultural University, China

Soil saline-alkalization is becoming a major constraint for agricultural production, seriously threatening global ecosystems and food security. An estimated 50% of total agricultural land will be salinized by 2050 due to factors such as low precipitation, high surface evaporation, poor cultural practices, over application of chemical fertilizers and so on [1]. It is important to note that approximately 60% of the saline soil has concurrent alkalization problems, conferred by high amounts of sodium carbonate (Na_2_CO_3_) or sodium bicarbonate (NaHCO_3_). Compared with pure neutral salt stress, combined saline-alkaline stress always results in higher cellular oxidative stress, more serious trophic ion imbalance, reduced osmotic adjustment capacity, and reduced uptake rates of essential nutrients [2]. According to the salt concentration and pH value of the soil, saline-alkaline stress can be classified into three levels: mild (salt content ≤ 0.3%, pH 7.1–8.5), moderate (salt content 0.3%–0.6%, pH 8.5–9.5), and severe (salt content ≥ 0.6%, pH 9.5) stresses [3]. With the progress of saline-alkaline tolerant crop breeding, the mild and moderate saline-alkaline soils are expected to be developed for crop production.

Our knowledge of how plants resist both salinity and alkalinity is vital for the modern molecular breeding of salt-alkali tolerant crops. Till now, how plants sense and respond to high salt stress has been

extensively studied [4], whereas the mechanisms of plants adapting to the high pH condition by alkaline stress remains largely unknown. Recently, the significance of understanding how plants adapt to alkaline conditions has been arousing increased attention. However, most studies on plant alkali stress response were restricted on elucidation of the regulatory mechanism of proton translocating ATPases in the plasma membrane (PM H^+^-ATPases), which establish proton gradients across PM to maintain the intracellular and extracellular pH and ion balance required for plant growth and stress response. For example, the activity of PM H^+^-ATPase AHA2 was inhibited by Ca^2+^ sensor SCaBP3/CBL7. Upon alkaline stress, the Ca^2+^ signal was triggered to relive the inhibition of SCaBP3 on AHA1 via both protein kinase SOS2-like 5 (PKS5)-dependent and -independent processes to help plants adapt to the alkaline environments [5]. Another Ca^2+^ binding protein, the C-terminal centrin-like domain containing protein 1 of *Triticum aestivum* (TaCCD1) confers plant alkali resistance by releasing the inhibition of the PM H^+^-ATPase TaHA2, which is suppressed by protein phosphatase TaPP2C.D1/8 under normal conditions [6]. In maize, the increase of cytosolic Ca^2+^ by salt-alkaline stress promotes proteasome degradation of an EF-hand Ca^2+^-binding protein ZmNSA1, there-by increasing the expression of two PM

H^+^-ATPases (*MHA2* and *MHA4*), and consequently enhancing the Na^+^/H^+^ antiporter SOS1 activity to facilitate root Na^+^ efflux to resist saline-alkaline stress [7].

Sorghum, the fifth main food crop in the world, originates from Sahelian belt in Africa where harsh environments make it more resistant than other crops to many abiotic stresses. Together with its relatively smaller size of genome and high qualitative parsed diploid genome sequence, sorghum has been recognized as a valuable cereal plant resource for exploring the genes acting in abiotic stresses’ adaptive ability. Sorghum *Alkaline tolerance 1* (*AT1*) gene was found to be closely correlated with alkaline stress tolerance via a genome-wide association study analysis with a large sorghum association panel collected from all over the world [8,9]. *AT1* encodes an atypical G protein γ subunit, and is homologous to rice *GS3* [10]. The full-length *AT1/GS3* negatively affects the alkaline tolerance in both sorghum and rice, while most of its naturally mutated forms result in a C-terminally truncated *AT1/GS3*, further enhancing the negative effect on plant alkaline tolerance [9]. Nevertheless, knockout of *AT1/GS3* conservatively confers to alkaline tolerance in four main monocot crops, including sorghum, rice, maize and millet. The detailed mechanism of *AT1/GS3* in plant alkaline stress response was also elucidated. *AT1/GS3* negatively regulates the phosphorylation of aquaporin PIP2s, which are responsible for H_2_O_2_ distribution under alkaline stress [9]. To evaluate the application of *AT1/GS3* gene, field tests on the sodic land were carried out. The nonfunctional mutants either generated by gene editing techniques

or identified from natural variations in sorghum, rice, maize and millet can increase crops yield and biomass by approximately 20% on salt-alkaline lands in Ningxia Hui Autonomous Region and Jilin province [9]. Therefore, this work revealed that the *AT1/GS3* gene would have great potential for breeding salt-alkali–resistant crops to grow on salt-alkaline soil for agricultural production.

Wheat, an important staple food crop, is widely cultivated around the world. Similar with other crops, salt-alkaline soil is also a big constraint to wheat productivity in multiple areas like northeast China. Thus, the function of the *AT1* homolog in wheat (*Triticum aestivum*, a hexaploid with genomes A, B and D) in aspects of salt-alkaline stress tolerance was explored. We generated *TaAT1* null mutants with all three copies of the *AT1* gene in the common wheat cultivar ‘Fielder’ using CRISPR-Cas9 gene-editing technology (Fig. 1A and B). Phenotype analysis showed that the survival rates of *TaAT1^ko^*-1 and -2 seedlings were 66.7% and 79.17% under 125 mM (104.2 mM NaHCO_3_ and 20.8 mM Na_2_CO_3_, pH 9.7–9.8) salt-alkaline treatment separately, which were greatly higher than the survival rate of wide type (WT) with 30% (Fig. 1C and D). Moreover, the relative plant height of the *TaAT1^ko^* plants was also higher than WT under alkaline stress (Fig. 1E and F). Thus, *TaAT1* knockout can greatly enhance salt-alkaline tolerance of wheat. To further investigate whether *TaAT1* uses a conserved mechanism as a defense against salt-alkaline stress as that in sorghum and rice, the ROS accumulation was measured in *TaAT1^ko^* lines by DAB and H_2_DCFDA staining. As shown in Fig. 1G–I, the intracellular H_2_O_2_ level in two *TaAT1^ko^* lines was much lower than WT under salt-alkaline treatment, consistent with *AT1^ko^*phenotypes of other species. There results indicate that genetic modification of *TaAT1* could efficiently improve wheat performance in salt-alkaline soil as *AT1/GS3* genes do in sorghum, maize, rice and millet with a conserved mechanism (Fig. 1J). Since the *AT1/GS3*-related technology works well in all the main mono-crops, genetic modification of *AT1/GS3* will greatly contribute to efficiently elevating the production ability of saline-alkaline land, and thus increase world food safety. Currently, there are ∼618 million hectares of sodic land around the world. According to our field data, we estimate that adaptation of only 20% of alkaline land to plant *AT1*-minus–related crops will generate at least 250 million tons of additional crop production per year around the world.

**Figure 1.**
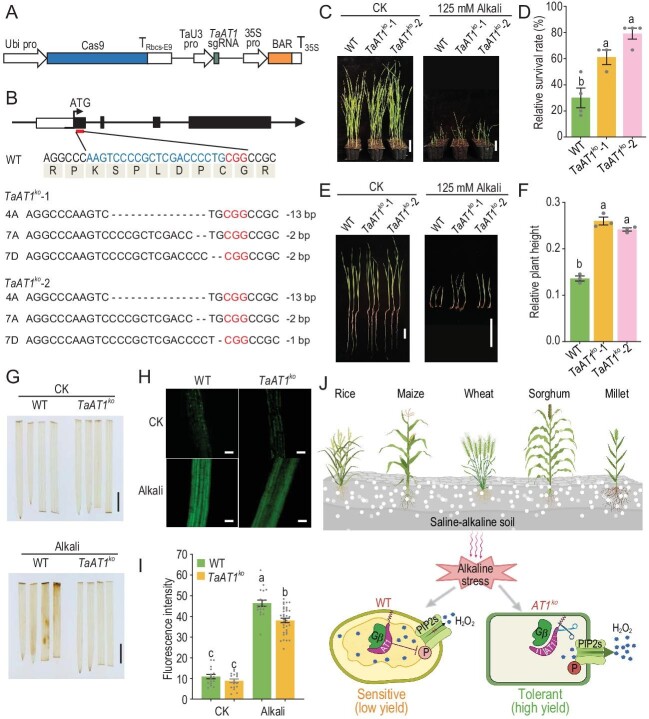
Gene-editing information of *TaAT1* in wheat and phenotype of transgenic plants in response to alkaline stress. (A) Structure diagram of the T-DNA structure in a CRISPR/Cas9 construct. (B) CRISPR/Cas9-induced mutagenesis of target genes. Target sequences and PAM sequences are indicated by blue and red text, respectively. The deletion bases are indicated in dashes. (C) Phenotypic analysis of WT and *TaAT1^ko^* plants under alkaline stress. Seeds were sown in soil without (CK) or with 125 mM mixed alkali. Photographs were taken on the 21^th^ day after seeds sowing. Independent experiments were repeated three times. Bars, 5 cm. (D) Statistical analysis of the relative survival rates of wheat lines in (C). Data are the mean ± SEM (n = 12 plants for each repeat) of three biological replicates. (E) Representative seedlings of WT and *TaAT1^ko^* plants with or without 125 mM alkali treatment on the 21^st^ day after seed sowing. Bars, 5 cm. (F) Statistical analysis of relative plant height of the wheat lines in (E). Data are the mean ± SEM of three representative plants in each line. (G) DAB staining of wheat WT and *TaAT1^ko^* leaves. Bar, 1 cm. Ten days seedlings treated with or without 250 mM alkali for 60 h were used for analysis. (H) H_2_O_2_ amount detection by a fluorescent probe (H_2_DCFDA) in the root of WT and *TaAT1^ko^* plants. Ten days wheat seedlings treated with or without 250 mM alkali for 48 h were used for analysis. Bars, 100 μm. (I) Statistical analysis of the H_2_O_2_ concentration in (H). Data are the mean ± SEM (n = 6 plants). In (H and I), *TaAT1^ko^* is *TaAT1^ko^*-2. In (D, F and I), statistical significance was determined by ordinary One-way ANOVA with Tukey's multiple comparisons test. (J) Working model of *AT1* in crops under alkaline stress.

For agricultural application, it might need the consideration of testing whether the integration of two or more saline and alkaline tolerance genes could further increase saline-alkaline tolerance in wheat and other graminaceous crops. The Na^+^ selective transporter *TmHKT1;5-A* from a diploid ancestral wheat relative *Triticum monococcum* has been reported to increase durum wheat grain yield by 25% on saline soils in field trials [11]. Since these genes are involved in different signaling pathways, we assume they have stack effects on salt-alkaline tolerance of the main graminaceous crops. Currently we have revealed that *AT1/GS3* genes play conserved and significant roles in the salt-alkaline stress response in five monocotyledon cereals, including rice, wheat, maize, sorghum and millet. Whether genetic modification of *AT1/GS3* orthologs can also enhance salt-alkaline tolerance in dicotyledon plants, such as soybean, remains to be studied. The other point we know is that many halophytes have a strong ability to survive in a saline-alkaline environment, such as sudangrass, seepweed, reed, salt horn grass and alkali grass. It is necessary to develop an effective strategy to explore conserved salt-alkali–resistant genes from these less studied halophyte plants, which may, in turn, benefit our studies on creating more salt-alkaline tolerance crops. In Arabidopsis, extracellular pH is sensed by plant cell-surface peptide-receptor complexes RGF1-RGFR and Pep1-PEPR in the root apical meristem region to regulate plant growth and immunity [12]. But how plants sense external pH and whether the mechanism that plants sense pH in alkaline soil is different from that in response to pathogen stress will be an important issue in future studies. Finally, since alkaline stress always co-occurs with saline stress, it remains challenging to elucidate the specific and common effect between saline and alkaline stresses.

## Supplementary Material

nwad075_Supplemental_File
