# Supplementary material for: Genetic modification of Gγ subunit AT1 enhances salt-alkali tolerance in main graminaceous crops
Source: Natl Sci Rev. 2023 Mar 23;10(6):nwad075. doi: 10.1093/nsr/nwad075 (PMC10171625; doi:10.1093/nsr/nwad075)
Supplement: nwad075_Supplemental_File [file nwad075_supplemental_file.docx]

**Genetic modification of Gγ subunit AT1 enhances salt-alkali tolerance in main** [**graminaceous crop**](javascript:;)**s**

Wenjing Sun^1,†^, Huili Zhang^2,†^, Sen Yang^2,†^, Lijing Liu^3,†^, Peng Xie^2^, Jian Li^1^, Yaoyao Zhu^1^, Yidan Ouyang^4^, Qi Xie^2,5,6*^, Huawei Zhang^1,*^, Feifei Yu^7,*^

^1^National Key Laboratory of Wheat Improvement, Peking University Institute of Advanced Agricultural Sciences, Shandong Laboratory of Advanced Agriculture Sciences in Weifang, Weifang, Shandong, 261325, China

^2^State Key Laboratory of Plant Genomics, Institute of Genetics and Developmental Biology, The Innovative Academy of Seed Design, Chinese Academy of Sciences, Beijing, 100101, China

^3^The Key Laboratory of Plant Development and Environmental Adaptation Biology, Ministry of Education, School of Life Sciences, Shandong University, 266237, Qingdao, China

^4^National Key Laboratory of Crop Genetic Improvement and National Centre of Plant Gene Research (Wuhan), Hubei Hongshan Laboratory, Huazhong Agricultural University, Wuhan, 430070, China

^5^National Center of Technology Innovation for Maize, State Key Laboratory of Maize Germplasm Innovation and Molecular Breeding, Syngenta Group China, Beijing, 102206, China

^6^University of Chinese Academy of Sciences, Beijing, 100049, China

^7^College of Grassland Science and Technology, China Agricultural University, Beijing, 100083, China

^†^These authors contributed equally.

^*^Correspondence: ffyu@cau.edu.cn (F.Y.), huawei.zhang@pku-iaas.edu.cn (H.Z.), qxie@genetics.ac.cn (Q.X.)

**SUPPLEMENTARY INFORMATION**

**ONLINE METHODS**

**Plant materials and growth conditions**

Seeds of wheat cultivar Fielder were grown in a greenhouse at a white light intensity of 250 mmol/m^2^/s under long-day conditions (16 h of light at 22–25°C/8 h of dark at 15–20°C).

**Construction of CRISPR**-**Cas9 plasmids**

The 19-bp fragment (Table S1) was selected from *TaAT1* coding sequence to synthesize guide RNAs (gRNAs) for editing *TaAT1* using CRISPR-Cas9 technology, annealed and cloned into *Bsa*I-digested pBUE411 vector. The expression of Cas9 was driven by the maize ubiquitin promoter and the expression of sgRNA was driven by TaU3 promoter.

**Wheat transformation**

Wheat transformation followed previously published protocols (Ishida et al., 2015). Briefly, we harvested immature grains from panicles approximately 14 days after anthesis and surface sterilized them with 70% ethanol for 1 min followed by with 1.2% (vol/vol) sodium hypochlorite for 10 min. After surface sterilization, we washed the immature grains three times with sterilized water and isolated immature embryos from them under a stereoscopic microscope.

We centrifuged the isolated immature embryos in liquid medium and then inoculated them with Agrobacterium carrying *TaAT1* editor. We transferred the embryos to co-cultivation medium with the scutellum side up and incubated them at 23°C in the dark for 2 days. Then, we excised the embryo axes, transferred them to callus induction medium and incubated them at 25°C in the dark. 5 days later, the embryos were transferred to selection medium with 5 mg/L phosphinothricin and incubated at 25°C in the dark for 3 weeks. After that, the calli was transferred to selection medium that contained 10 mg/L phosphinothricin at 25°C in the dark for another 3 weeks, and then to regeneration medium containing 5 mg/L phosphinothricin and incubated at 25°C under continuous illumination (68 μmol/m^2^/s) for 2 weeks. We finally transferred the regenerated plants into rooting medium that contained 5 mg/L phosphinothricin to induce roots. The rooted plants were transplanted to soil in a greenhouse to grow for 3-4 months, and progeny seeds were harvested from them.
**Genotyping**

To genotype the T_0_ transgenic lines and their progenies, genomic DNA was extracted from leaves to amplify the targeted sequences of sgRNAs for Sanger sequencing Then, the mutagenesis ratio was analyzed based on the sequencing results using Dsdecode (Liu et al., 2015). The PCR primer sets are listed in Table S1.

**Phenotypic evaluation**

Seeds for phenotypic evaluation were harvested at the same time and in the same place, and kept under the same conditions. 125 mM alkali treatment was selected as the most appropriate concentration for identification of wheat alkali stress phenotype. The detailed working concentrations of NaHCO_3_ and Na_2_CO_3_ in 125 mM alkali are 104.2 mM and 20.8 mM, respectively. The growth phenotype, relative survival rate and relative plant height were recorded under 125 mM alkali stress for 3 weeks.

**Reactive oxygen species (ROS) and H_2_O_2_**

The formation of peroxides in wheat leaves was studied by 3,3'-diaminobenzidine (DAB) staining (Jambunathan., 2010). Seedling leaves of Control (CK) and alkali treatment (250 mM) were soaked in 10 mL of dye buffer (50 mM Na_2_HPO_4_ and DAB). After vacuuming, the samples were incubated overnight at room temperature in the dark. The samples were transferred to an eluent (anhydrous ethanol: glacial acetic acid: glycerol = 3:1:1), and boiled for 10 minutes to remove chlorophyll, and then the sample was photographed under the same conditions.

To detect cytosolic ROS levels in plant root tips of *TaAT1*-related genetic materials, OxyBURST Green H_2_DCFDA dye was used (Monshausen et al., 2007). Ten-day wheat seedlings grown under normal conditions were treated for 48 h with or without 250 mM alkali. Then, the lateral roots of the seedlings were collected, cleaned and gently transferred to 3 cm round dish. The H_2_DCFDA was dissolved in dimethyl sulfoxide (DMSO) to 10 mM, and then diluted with sample buffer (10 mM Tris-HCl (pH 7.2), 50 mM KCl) into a final concentration of 50 μM. The sample was soaked in 0.01% Tween 20, vacuumed for 30 minutes, rinsed twice in distilled water, and then washed with a wash buffer (10 mM Tris-HCl pH 7.2 and 50 mM KCl). Incubate with 50 μM H_2_DCFDA staining solution at room temperature dark environment for 6 min. Remove excess dye twice with distilled water. The samples were prepared using a Zeiss LSM 510 confocal microscope, excited at 488 nm and emitted at 530 nm. In order to compare fluorescence intensity, all parameter axes under different experimental conditions were fixed at the same time and analyzed under confocal microscope under the same setting. Five to twenty lateral roots were selected for each sample, and 1-3 visual fields were selected for each lateral root for imaging. In order to quantify intracellular ROS accumulation at the level of single root cells, three representative root cells were selected from each visual field for statistical data. The mean value of fluorescence intensity was recorded for each repeat.

**REFERENCES**

Ishida, Y., Tsunashima, M., Hiei, Y., Komari, T. (2015). Wheat (*Triticum aestivum* L.) Transformation Using Immature Embryos. In: Wang, K. (eds) Agrobacterium Protocols. Methods in Molecular Biology, vol 1223.

Liu, W., Xie, X., Ma, X., Li, J., Chen, J., Liu, Y. (2015). DSDecode: A Web-Based Tool for Decoding of Sequencing Chromatograms for Genotyping of Targeted Mutations. Mol Plant. Sep;8(9):1431-3.

Jambunathan, N. (2010). Determination and detection of reactive oxygen species (ROS), lipid peroxidation, and electrolyte leakage in plants. Methods Mol. Biol. 639, 292-298.

Monshausen, G., Bibikova, T., Messerli, M., Shi, C., Gilroy, S. (2007). Oscillations in extracellular pH and reactive oxygen species modulate tip growth of Arabidopsis root hairs. Proc. Natl. Acad. Sci. USA 104, 20996-21001.

**Table S1. List of primers used in this study.**

| ID | Primer sequence (5’-3’) | |
| --- | --- | --- |
| \| sgRNA-F \| GGCAAGTCCCCGCTCGACCCCTG \| \| --- \| --- \| \| sgRNA-R \| AAACCAGGGGTCGAGCGGGGACT \| \| TaAT1-4A-F \| CTTGGCTAACCCTACCCACTACCC \| \| TaAT1-4A-R \| CTGTGTGCTGGTAACAGACTCTAATACCTACTAC \| \| TaAT1-7D-F \| GTCTGCTAGATTGATCGTTCATTCACTCC \| \| TaAT1-7D-R \| GGTAGCGGACTTTAGATACCTATAATAGAGTACTCATAC \| \| TaAT1-7A-F \| CATTCCATTGATGACGCTCTCTGGC \| \| TaAT1-7A-R \| GGAAGTGACAACTAAACTGTGTGCTGG \| | |  |
